# CLM App: Interlamellar Distance of Pearlite via CLM Revisited and Automated

**DOI:** 10.3390/ma19030635

**Published:** 2026-02-06

**Authors:** Martin Zouhar, Šárka Mikmeková, Jan Hovjacký, Petra Váňová

**Affiliations:** 1 Institute of Scientific Instruments of the Czech Academy of Sciences, v. v. i., Královopolská 147, 612 00 Brno, Czech Republic; sarka@isibrno.cz; 2 Faculty of Materials Science and Technology, Vysoká Škola Báňská-Technical University, 17. listopadu 2172/15, 708 00 Ostrava-Poruba, Czech Republic; jan.hovjacky@signumcz.com (J.H.); petra.vanova@vsb.cz (P.V.)

**Keywords:** quality assessment, pearlite spacing, electron microscopy, microstructure, automated image analysis, Python software

## Abstract

Pearlitic (stainless) steel is used in automotive, aerospace, and other industries where high strength, hardness, and wear resistance are required. Its quality control can be performed using mechanical tests or by examining the lamellar microstructure, namely, determining interlamellar distance. One of the related approaches is the circular line method (CLM). This paper reviews the challenges to automate employment of the CLM using custom Python code in order to reduce human time costs during image-based quality assessment of pearlite. The goal is to perform intersection counting automatically once the human operator has configured the application and selected the locations of measuring circles. Performance assessment using manually processed data from some 465 images is performed. We divide the imaged pearlite microstructures into different “types” when the code performs well or, respectively, not so well. We conclude with possible extensions of the work presented here.

## 1. Introduction

The topic of establishing interlamellar distance in pearlitic steel from microstructure metallographic images is a long-standing one. Ref. [1] states that the topic has been explored at least since the 1920s and is still alive, as demonstrated by the appearance of fairly recent papers, e.g., Ref. [2]. One of the reasons for this longevity is the industrial importance of this two-phase lamellar structure consisting of ferrite (α-Fe) and cementite/carbide (Fe_3_C). Another contributing factor is that the development of scanning electron microscopes (SEMs) has allowed for an increase in magnification and in the resolution of steel microstructure images. This, in turn, has made related image analysis feasible in the case of finer pearlitic structures which are hard to resolve in light optical microscopes.

Despite the imaging technology advances, the work remains mostly tedious, comprising the straightforward analysis of visual data. There have been attempts to automate it [3,4] using custom code. One of the problems with full automation is selecting regions of interest (ROIs) that contain a reasonably regular distribution of the lamellae or subtracting “noise” corresponding to irregularities from regions among individual pearlite colonies. With the rise in applications of machine learning (ML) and so-called artificial intelligence (AI) applications, one may consider the use of such tools to assist in the selection of ROIs and in image segmentation. Image segmentation can be performed by classifying the two most relevant phases (see, e.g., Ref. [5]) using ML, or, more specifically, a support vector machine, to achieve a similar goal. Nevertheless, image processing has already been recognized as a well-established data science practice before the ML/AI boom. We strongly believe that it provides the sufficient tools to deal with the task at hand.

The approach of Ref. [4] (the authors of which kindly provided custom MATLAB software (The MathWorks, Inc., Natick, MA, USA) code (working with MATLAB version R2018b), shown in their Supplementary Materials) attempts to tackle both; it uses the good periodicity of the lamellar structure (especially true in case of an ROI singling out a regular pearlite colony) and employs discrete Fourier transformation (DFT). Analyzing the results of the DFT provides the (apparent) interlamellar distance.

This paper focuses on the CLM method. The CLM method, together with the data-processing algorithm and its code-implementation approach, is concisely formulated. The performance of the code is evaluated on a manually processed dataset. Furthermore, a comparison with the MATLAB DFT-based code is included.

## 2. Materials and Methods

### 2.1. Circular Line Method (CLM) in a Nutshell

**Figure 1:**
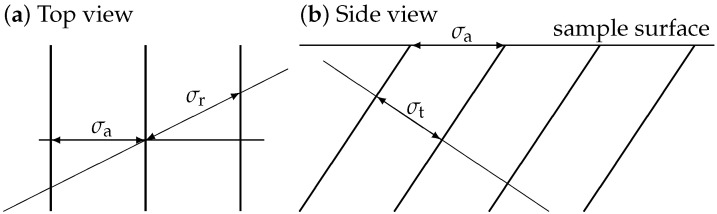
Schematic skeleton representation of an ideal lamellar structure cross-sectioned by the sample surface plane. (**a**) Top view. The surface plane contains a randomly placed intercept line, leading to $\sigma_{r}$, and a line perpendicular to the 2-dimensional sections of the lamellae blocks leading to apparent distance $\sigma_{a}$. (**b**) Side view. Finally, there is the sought-for true distance $\sigma_{t}$ which is derived from the full 3-dimensional structure and not its 2-dimensional section. The apparent interlamellar distance $\sigma_{a}$ is also indicated.

Consider a periodic 3-dimensional lamellar structure.A microscopic image represents a cross-section of the 3-dimensional structure with the plane defined by surface of the sample. Determining the interlamellar distance σ from such a cross-section correctly requires keeping the relation of the cross-section to the 3-dimensional structure in mind. This leads to a practical necessity to distinguish among several kinds of interlamellar distance, as illustrated in Figure 1.

The different kinds of the interlamellar distance σ are random (“r”), apparent (“a”), and true (“t”). The first two are determined from the (imaged) sample surface plane, which, in general, is not perpendicular to the planes of the lamellae, which means that these two values overestimate the true one. This immediately follows from the fact that each of these two values corresponds to a hypotenuse in a suitably chosen right-angled triangle. Figure 1a shows a top-view, i.e., to the sample plane. It is clear that for any angle, the random intercept line leads to an interlamellar distance larger than or equal to the apparent value, $\sigma_{r}\geq \sigma_{a}$. A similar conclusion can be drawn from Figure 1b for the apparent and true interlamellar distances, i.e., $\sigma_{a}\geq \sigma_{t}$.

For the sake of completeness, let us note that corresponding variants of (nearest) edge-to-edge distance δ can be defined accordingly.

The statistical mean values of these center-to-center distances σ satisfy the following relation (see Equations (1) and (2) in Ref. [3]):

(1) $${\bar{\sigma }}_{t}=\frac{\pi }{4}\cdot {\bar{\sigma }}_{a}=\frac{1}{2}\cdot {\bar{\sigma }}_{r},$$

where the mean is indicated by an over-bar.

The CLM method is fairly simple and it consists of several steps described in the following.

1. Draw a measuring circle of a known real-space diameter *d* (m) in the field of view part of the microscopic image. As each pixel has a given real-space size, its value depending on magnification, this effectively means converting the value of diameter from real-space to its counterpart in pixel-coordinates.
2. Count all intersections of the lamellae with the measuring circle *n*; counting the number of intersecting lamellae objects $n_{\mathrm{obj}}$ can also provide useful information.
3. Calculate one of the (center-to-center) interlamellar distances σ; its value is directly proportional to the ratio d/n or $d/n_{\mathrm{obj}}$.
  (2) $$\sigma_{r}=\pi \frac{d}{n},\;\sigma_{a}=\frac{d}{n_{\mathrm{obj}}}.$$
4. Statistical processing of the data to arrive at the (batch or single file) mean value $\bar{\sigma }$.

The processing is, therefore, relatively straightforward once the object is segmented into individual lamellae and the background components. The implicit assumption is that the circle diameter *d* is sufficiently large for it to enclose a rather large number of lamellae but sufficiently small to minimize occurrences of irregularities, e.g., among different pearlite colonies.

First, the CLM is applied to the sample surface plane. Second, the circle segments defined by a pair of nearest neighbor intersection are rather randomly oriented with respect to the lamellae colony. This means that the σ derived in Equation (2) is the “random” interlamellar distance (compare also with Equation (9) from Ref. [3]).

Thus, there are two opposite demands on the diameter *d* of the measuring circle that have to be balanced for each given microstructure image. In order to cover a reasonable number of random directions of the intercepting segments of the measuring circle, its diameter *d* has to be large, i.e., it is desirable to enclose as many lamellae as possible. This, on the other hand, increases the chance of including defects or adjacent pearlite colonies (which may have a different orientation). Furthermore, it leaves less space for other measuring circles with minimal overlap to provide enough statistically independent data coming from more circles located in the same image. For example, the number (respectively, the diameter *d*) of measuring circles in a given microstructure image may be restricted from below (respectively, from above) by standards providing a guide for applying CLM to a pearlitic steel for a given industry segment.

### 2.2. Manually Processed Dataset

The performance of the yet to be discussed automation is tested on manually processed data (analysis by J.H. and P.V.) of a pearlitic steel microstructure. The steel was imaged using a Quanta FEG microscope (Waltham, MA, USA) equipped with an Everhart-Thornley Detector (ETD) operating in the secondary electron mode. The advantage of secondary electrons is that they “enhance” the topography of the sample.

The image dataset corresponding to manually processed data consists of 465 pairs of microscopic images total and an accompanying MS Excel spreadsheet. Relevant data in the spreadsheet consists of values of intersection counts for a given image file (without assignment to a specific measuring circle) and derived interlamellar distances. The magnification ranges from 25,779× through 30,000× (the most frequent with 460 occurrences in the original microscopic images) up to 32,715×. The value 30,000× represents a reasonable compromise ensuring good image quality. The images are stored in JPG format.

**Figure 2:**
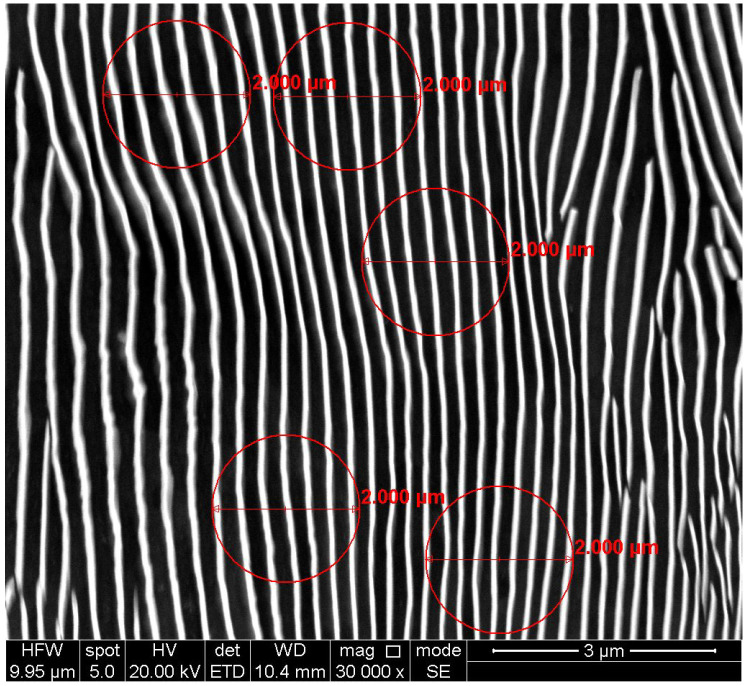
An example SEM-ETD image from the manually processed dataset; the dark background corresponds to the ferrite regions, and bright lamellae consist of cementite. The red-colored overlay over the original microscopic image consists of measuring circles, each with an accompanying label stating diameter size of 2.000 µm. The contrasting overlay allows relatively easy automatic detection of the circle position.

Each image pair contains the original microscopic image and the image with measuring circles being marked; see Figure 2. These pairs correspond to 10 different samples prepared using various technological methods/processes, including, but not limited to, different duration of cooling.

As a result, the samples exhibit different microstructures, namely, interlamellar distance and thickness and average length of the lamellae. Furthermore, each sample is split into four batches, each batch corresponding to images from a different regions of the sample. A single batch contains approximately a dozen images.

The dataset has been meticulously rechecked (manual-part by P.V. and code-part by M.Z.) to avoid any inconsistencies. These may include corrupted or missing original microstructure images or circles slightly overflowing the field-of-view. The human evaluator/operator can extrapolate and “fill in” the missing parts of both the circle and lamellae as needed. This, however, is not possible for a code that operates strictly with data in the field of view which justifies the removal. On top of that, automatic circle detection misperformed on few files with measuring circles. These have been omitted in order to keep the data comparison as reproducible as possible on the code side.

**Figure 3:**
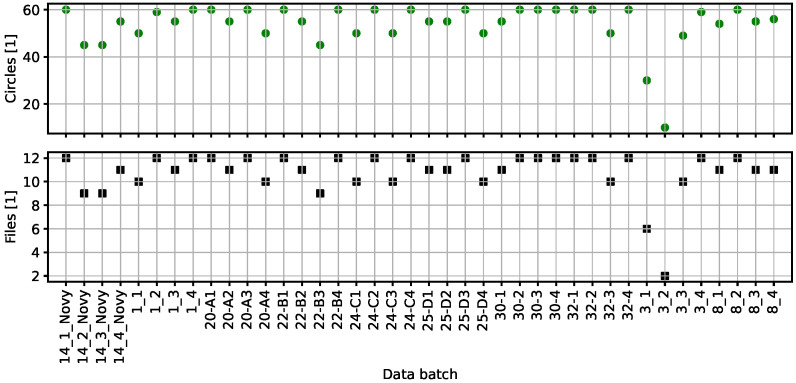
Overview of the 40 batches available in the verified/reduced manually processed data—file count (**bottom**), total 431, and total circle count in a given batch (**top**). The naming convention reflects the directory structure of the dataset and each name consists of two items; integer sample identification followed by a batch name (the separator being either an underscore or a dash).

As a result, only 431 microscopic images, corresponding to 10 samples and 40 batches, were used in the Python code (version 0.0.6) benchmarking. A typical batch contains from 9 to 12 images; the total number of measuring circles is not less than 39 and it does not exceed 62. We believe that some forty circles represent a large enough statistical sample for this kind of processing. There are two batches related to sample no. 3 with the image count rather significantly reduced due to the data reduction described above. The information on batches is visualized in Figure 3.

We note that the MS Excel sheet contains data-blocks corresponding to circles in a given file without any assignment of the actual circles, present in the corresponding image, to the individual records. This, of course, was irrelevant for the manual processing.

Furthermore, the naming convention of the as–were original microstructure images and those with measuring circles marked is not always consistent.

**Figure 4:**
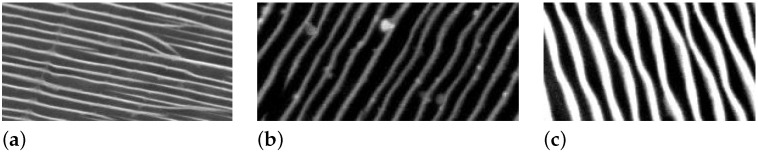
Visual examples of regions with more complex features, pearlite colonies containing irregularities of various kinds. (**a**) Strong background halo, scratch, bended lamellae, branching/merging lamellae. (**b**) Mostly kinky and/or wavy lamellae. (**c**) Irregularly wavy lamellae. The ruler is omitted as this image serves merely to qualitatively illustrate some features. The data were not processed further beyond cutting the displayed regions using GIMP software (version 3.0), exporting to the same file format with maximal quality setting and slightly resizing the cuts to achieve similar height.

The “quality” of the as-imaged microstructures differ. Some regions, or even whole fields of view, are formed by high-contrast, bright, regular, well-separated lamellae on the backdrop of a dark background.This, however, does not apply to the whole dataset utilized; examples of more complex features in the pearlite colonies are displayed in Figure 4. The richest representative is shown in Figure 4a. First, a background grayish halo is present everywhere, leading to lower contrast between the lamellae and the background. This is not a serious issue if the background is fairly uniform and its intensity is sufficiently well separated from the intensity of the lamellae. If not the case, the local segmentation into lamellae might perform worse. Second, there is a scratch-like defect stretching from near the left-bottom corner both upwards and towards the right-hand-side direction. Third, some lamellae are bent. Fourth, the image exhibits merging of several lamellae or branching of some lamellae, especially in its right-hand-side half.

Figure 4b contains lamellae with strong kinks, sometimes even with strong-intensity spots centered rather well within the lamellae, or lamellae that are wavy. Furthermore, the leftmost incomplete lamella from the top has two notable features—its end is single-sidedly tilted (towards the right) and the upper part of the lamella is seemingly joined to another lamella by a fairly large bullet-like defect.

### 2.3. Interlude: Tackling Object Distance in Image Data of a Layered Structure

Let us disregard CLM for a moment and consider a daring approach—segment the image into individual lamellae and determine their distance directly.

This processing assumes a certain way of segmentation, i.e., specification of lamella boundary. Is it a threshold value for intensity gradient or simply for intensity? How exactly would the latter be related to the intensity values in the image is not unique as there are several competing well-established thresholding methods. The choice of the boundary definition affects the position of the boundary. One can argue that the effect would be only a few pixels and it could perhaps be neglected. Or, one could use some preliminary segmentation with an additional step selecting maximal intensity leading to a “skeleton” representation, a single lamella being represented by a 1-dimensional curve C.

The distance would have to be determined locally, i.e., for each pixel from a given reference lamella, find the closest pixel from all the other lamellae and keep only the smallest value that should correspond to the nearest-neighbor lamella. The resulting computational overhead of the aforementioned robust finding of a single value could be justified by the reduced cost of human effort, but, still, this approach seems to be an overkill. One could use a locally perpendicular line and find the nearest intersection and do this along the whole lamella; this would determine the local value of apparent interlamellar distance $\sigma_{a}$. This could be repeated for all other remaining lamellae and then simply handle the overcounting due to the inclusion of the local distance of two lamellae $C_{i}$ and $C_{j}$ twice—the first one, *i*, from the other, *j*, and the other one, *j*, from the first one, *i*. On the one hand, the local-perpendicular direction is computationally less demanding than trying all directions. On the other hand, it requires construction of the local perpendicular direction. This may not be easy if there are local irregularities such as any of the following—kinks, asymmetric thinning or widening, bending (e.g., due to scratches of the sample surface) or lamellae being wavy, or sharp tilted ends of the lamellae (see Figure 4 and related discussion in the text). Such defects could cause misalignment of the local perpendicular direction and affect the values of $\sigma_{a}$.

One could represent the real lamellae by some fictitious idealized “straightened” out smoothened versions. Now, consider a pearlite colony whose lamellae locally highly deviate from a straight line (wavy “oscillations” or an overall bending) though they are straight lines “overall”. Should such a colony be replaced by a fictitious new one with its members being idealized straightened versions of the original ones? This will not work well if the ROI contains mostly uniform bending instead of wavy oscillations.

Furthermore, interlamellar spacing along the perpendicular direction is influenced by the sectioning plane effect [6].

Last, but not least, consider a branching lamella (Figure 4a)—a single trunk/parent lamella apparently forks into several branches/children lamellae. How is this corner case to be treated? Should the distance among child lamellae be calculated as well? Let us note that our algorithm attempts to do so. If so, how can such a branching be automatically detected, and how can the split of the trunk and its branches be performed?

With these issues to tackle, we conclude that the daring approach is hard to implement correctly, starting from the definition of the boundary of a lamella and dealing with the corner cases described above and illustrated in Figure 4. This might not be a critical issue as the amount of data to be acquired is large. As a result, these corner cases may be relegated to marginal contributions only, not significantly affecting the average value. Still, the computational power and coding effort required to collect all these data (nearest neighbor distances along lamellae) is significantly larger than that of the well-established CLM approach. Therefore, we conclude that the above suggested daring approach is to be abandoned in favor of a much simpler CLM implementation that keeps in mind the lessons learned in the above discussion.

### 2.4. A Tale of Two Approaches

We propose two approaches and discuss their challenges:

(A1) Geometric—single lamella treated as an idealized rectangle-like object, at most two crossings with the measuring circle.
  The number of crossings is determined from the positions of endpoints of this idealized lamella with respect to the measuring circle.
  This geometric approach leads to undercounting in general, as the desired result is that each child of a branching lamella can contribute to the (manual) crossing count. However, this is no longer guaranteed as the imposed restriction to the maximal number of intersections of a single object. To be more precise, the single object here means a single connected component characterized by a high value of intensity in the thresholded and segmented image. If branching lamellae are not correctly split into several objects, the undercounting occurs.
  A partial remedy is that the segmentation into individual lamellae occurs locally in the square-shaped vicinity of the measuring circle processed (padding of the measuring circle can be a free parameter). The local segmentation will detect each child lamella as an individual lamella, provided that the branching occurs outside of this immediate neighborhood. A full workaround is to instruct the end user not to select regions where branching lamellae occur in such a way that the branching point is excluded from the square enclosing the measuring circle.
(A2) Image-data—count-connected components of the crossings of the measuring circle with the individual lamellae.
  The restriction of at most two crossings for a single lamellae from the approach (A1) is not applied here. As a result, this “image-data” approach tends to overestimate the actual counts. The reason for that being that noise and finite pixel-size effects (e.g., circle is represented by a collection of pixels that form a ragged curve) cause small spurious artifacts that are incorrectly counted as valid crossings. A partial remedy is to smear the image before the segmentation; this may reduce these artifacts. Furthermore, one could reduce the number of spurious artifacts by requiring a valid crossing to have a minimal size of at least *s* pixels, where *s* is an unsigned integer. It can be set as a fixed pixel size (or alternately as a fixed real-space size) or as a relative number (fraction of estimated thickness of a given lamella). This, however, leads to optimizing for a given test image dataset and its variations across different types of sample images (thick, well-separated, line-like lamellae vs. thin, curly lamellae). The most flexible solution would be a configurable parameter with a default value and suggestions on how to modify it for a given pearlite colony type which, at the end of the day, would be the responsibility of a human operator. This, however, does not handle overcounting due to locally (highly) non-linear lamellae; see Figure 4c.

**Figure 5:**
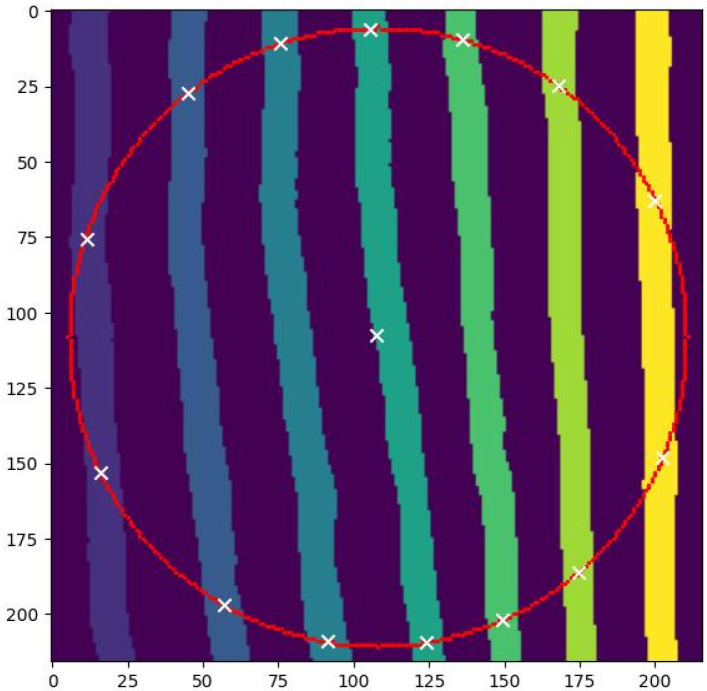
Image depicting the inner workings of the algorithm starting with step (S5) in the approach (A2). A vicinity of a single measuring circle (color: red) from Figure 2 is considered. The different shadings (colors: from blue through green to yellow) distinguish individual lamellae (respectively, connected components in the corresponding region of the original image). Crosses (color: white) indicate “mean” positions of connected components of crossings of the lamellae with the measuring circle. The near-center cross corresponds to all the dark background parts and it does not contribute to the sought-for intersection count *n* (the current value of which is 14).

With the above considerations in mind, we can present a high-level overview of image processing with the measuring circles already selected. The steps performed to arrive at the results are the following:

(S1) Load the image data as grayscale (possible to invert intensity).
(S2) Separate data bar, if present, from the field of view.
(S3) Perform global thresholding of the image (method configurable).
  Thresholding improves separation of the lighter lamellae from a dark background. If there is a non-dark halo surrounding the lamellae, careful selection of the thresholding method may be needed.
(S4) Smear the image (blurring window size configurable).
  This helps to reduce effects of noise, e.g., grainy images with lower intensity of the lamellae, and tiny artifacts.
(S5) For each measuring circle:
  (a) Perform local segmentation into individual lamellae in the vicinity of the measuring circle.
    The local segmentation may help to avoid occurrences of a branching point. Its presence in the ROI enclosing the measuring circle would lead to a branching lamellae and all its children being considered as a single object by the code. This would decrease the number of lamellae intersecting the circle, thus affecting the “geometric” approach (item no. (A1) in Section 2.4).
  (b) Count intersections and store the final intersection count *n*.
    A count correction can be implemented as discussed in the presentation of the two approaches. For example, drop contributions of objects too small (likely defects or visible ends of lamellae which are predominantly located out of the field of view) allow for, at most, two intersections for a single object (the geometric approach vs. branching lamellae), etc.
  The intermediate image processing is visualized in Figure 5.
(S6) Post-processing the intersection counts, values of *n*, is performed to arrive at the interlamellar distance σ.

Let us note that none of the above methods is fully immune to the isolated point-like defects that cross the measuring circle. A significantly more complicated logic could be devised. First, check the pearlite colony or colonies in the whole image and determine the average “length” of lamellae not ending on the edges of the field of view (may be negatively affected by the branching lamellae). Second, eliminate isolated point-like defects (objects too short) not at the edge of the field of view (i.e., transforming the underlying data-image). This would still leave the point-like defects at the edge of the field of view, which could be handled by requesting the user not to place the measuring circles near these edges. Of course, one could consider implementing ML/AI through a neural network trained to classify the objects into a standalone lamella, a branching lamella, a point-like defect, etc. However, any inclusion of ML/AI would complicate code employment for the end user (additional dependencies and most likely an HW-specific installation/compilation), and a software as a service (SaaS) solution would be more meaningful in this case. Anyway, this is beyond the scope of the presented algorithm and its implementation.

We conclude this general section by commenting on pearlite colonies’ structural characteristics, other than interlamellar distance, that are available as a byproduct of the above-described algorithm. As we have the microstructure segmented into individual lamellae and the background, we can calculate the ratio of the surface corresponding to the background and lamellae. One could, in principle, attempt to determine the thickness of the individual lamellae. This is not a simple task due to two reasons. First, Figure 4c shows lamellae with varying thickness, i.e., lamella thickness is a local quantity. Thus, a single number would provide incomplete information in the form of extremal value or some form of average. Second, if the single segmented object is a branching lamella (discussed already in Section 2.3), then its local thickness determination as the most distant pixels along the locally perpendicular line would fail. It would provide the outer distance between the outermost child branches of the segmented object in its branched region, i.e., severely overestimating the thickness of the individual (though unresolved as such) child branches.

### 2.5. Implementation

The aforementioned algorithm has been implemented in a Python code by one of the authors (M.Z.) and it is tentatively titled “CLM App”. It can be called from the command line with several “expert” options and it provides an accompanying GUI. The code utilizes several non-standard Python libraries (see Appendix A) to achieve its goal, and its availability has recently been presented to the materials science community [7].

**Figure 6:**
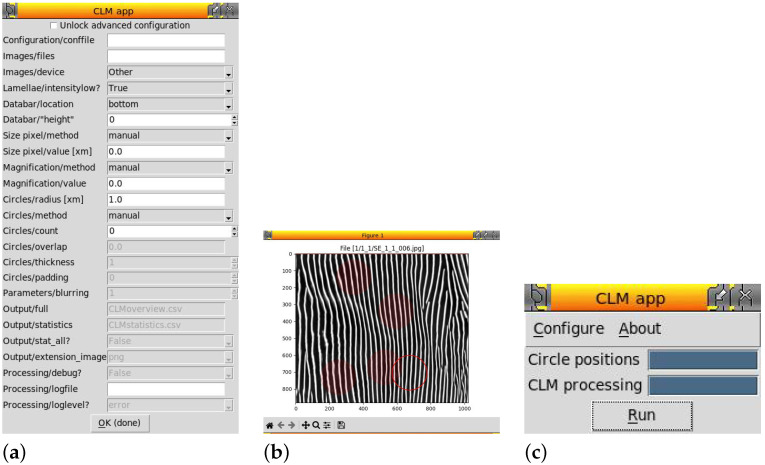
Screenshots of the various windows of the Python code CLM App, version 0.0.4 (semantic versioning). Left to right: (**a**) configuration window; (**b**) selecting circle positions (plot produced by the Matplotlib library [8], version specified in Appendix A)—the already selected positions are indicated by transparent dimmed red-filled circles and the current cursor position is marked by a non-transparent boundary of a red circle; (**c**) main window with the processing completed as indicated by both progress bars being filled.

The various windows of the TkInter GUI are displayed in Figure 6. TkInter, a standard library of Python, was selected because the licensing was the least limiting when compared to other Python GUI frameworks.

The input files are microscopic 8-bit images. Benchmarking data discussed in the next sections are produced by SEM-ETD imaging. In the case of using SEM-CBS, the image intensity is essentially “inverted”. In order to account for different detector types, the data processing includes an intensity inversion switch to ensure that the lamellae of interest are represented by bright regions.

Pixel sizes of each image can be automatically detected from metadata (in the case of TIF(F) images), from optical character recognition of the data bar (provided that an optional dependence Google tesseract OCR [9] is installed), or it can be manually set to a constant value for all batch-processed images. The aforementioned automatic detection is available only in the case of several electron microscope devices—test images that were available to us—which are supported as of this moment, but adding more is not a difficult task.

This code produces several CSV output files and it has built-in logging. The CSV files are human readable, easy to process by other code, and easy to import into spreadsheet applications such as MS Excel and LibreOffice Calc.

The code can be distributed in the form of a binary (and accompanying additional files) using code-freezing. This approach makes it fairly portable and it minimizes the installation of dependencies on the on-premise PC to a bare minimum. Of course, the code-freezing is performed on the target OS, or its reasonable equivalent, which has to be available to us. An accompanying manual explaining the configurable parameters and outputs has already been written.

## 3. Results and Discussion

The MS Excel spreadsheets with manually processed data provide intersection counts without explicit association with corresponding measuring circles. Because of this ordering uncertainty, the deviation from the manual data is calculated as follows—we minimize the sum of absolute values of individual differences of the two intersection datasets across all possible permutations *p* of records from one of the datasets for a given microscopic image. This provides intersection count deviation for a single image, $\Delta_{n:1}$. We also define a relative deviation $\delta_{n:1}$ as follows.

(3) $$\Delta_{n:1}=\min_{p}\left\{\sum_{i=1}^{N}\left|n_{i,\;\mathrm{manual}}-n_{p(i),\;\mathrm{code}}\right|\right\},\;\delta_{n:1}=\frac{\Delta_{n:1}}{\sum_{i=1}^{N}n_{i,\;\mathrm{manual}}},$$

where *N* labels the number of measuring circles within the considered microstructure image.

These quantities are the measure of performance for a single image. An initial test run used the Otsu threshold and the minimal non-trivial blurring window size (see Appendix B for more details). A comparison of the two lamellae treatment methods introduced in Section 2.4 reveals that there are data for which the first performs better than the second, and vice versa. The best performance is achieved on images with thick, well-separated, fairly linear lamellae, and the relative deviation does not increase over a few units of percent. The deviation increases up to tens of percent as the lamellae become thinner and locally highly curved.

However, the most significant quantity is the interlamellar distance σ. Therefore, we also compare mean values of the random interlamellar distance ${\bar{\sigma }}_{r}$ in each of the batches. Specifically, we shall focus on relative difference with respect to the manual data:

(4) $$\delta_{\bar{\sigma }}=1-{\bar{\sigma }}_{\mathrm{code}}/{\bar{\sigma }}_{\mathrm{manual}}.$$

The statistical mean values of interlamellar distance appear in division. Hence, the scale factor, relating the three alternate distances in Equation (1), cancels out. Consequently, any mean interlamellar distance can be used in Equation (4).

**Figure 7:**
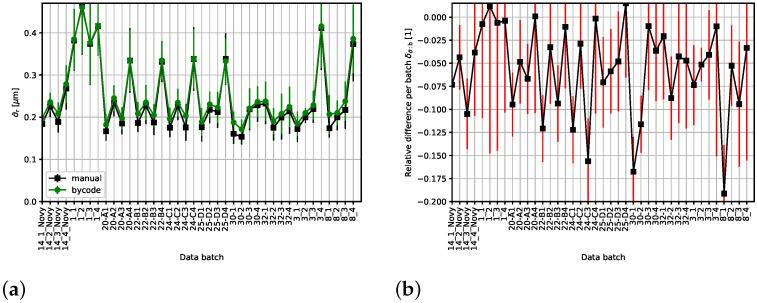
Best overall results of mean interlamellar distance $\bar{\sigma }$ for the 40 batches used in the benchmarking of the code (see Figure 3). The code produced values were obtained with the image-data-based approach and Otsu threshold. (**a**) The manual processing data points are represented by squares (black) and the code results are marked by circles (green). (**b**) The relative difference $\delta_{\bar{\sigma }}$ of the $\bar{\sigma }$ per batch, i.e., $\delta_{\bar{\sigma }:b}$, with respect to the manually processed data. Error bars (red), complementing the data-points (black), are derived from those in the preceding image segment.

We tested both the geometry-based and image-based approaches discussed in Section 2.4 using various thresholding methods. The relevant processing parameters, specific to the Python code, are presented in Appendix B. The best overall results—with minimal cumulative relative differences of $\bar{\sigma }$—are obtained in the case of the image-based method and Otsu thresholding. They are displayed in Figure 7. The error bars are equal to Figure 7a, or derived from Figure 7b, with standard deviation as implemented in the NumPy library [10]. We note that the “isodata” and “mean” thresholds (all of the three available in SciKit-Image [11] used in our code; see Appendix A) perform similarly well on average.

Let us recall Equation (2) stating that σ is proportional to the inverse value of the intersection count *n*. Whenever the relative difference $\delta_{\bar{\sigma }}$ displayed in Figure 7b is positive, the code, on average, overestimates the intersection count *n*. Conversely, a negative relative difference $\delta_{\bar{\sigma }}$ indicates code underestimating the intersection count *n*.

The latter is typically the case, as demonstrated by the data displayed in Figure 7. This is a rather surprising conclusion, contradicting the simple expectation based on discussion of the image-based approach (A2) in Section 2.4.

The highest deviation, exceeding 10%, with respect to the manual processing occurs in the case of the following batches: 8_1, 30−1, 24−C3, 24−C1, 22−B1, 30−2 and 14_3_Novy, as is clearly seen from Figure 7b.

Exploring the corresponding image dataset, the following is revealed:

- 8_1: The images are somewhat “grainy” with lower contrast. The lamellae have somewhat less intensity and they are sometimes surrounded by a background halo of varying intensity. There are few regions with the lamellae being very close. This, and the presence of the halo, complicates proper segmentation, leading to several lamellae being treated as a single, connected, large object. This apparent “blobbing” leads to underestimation of the number of intersections.
- 30−1: Approximately half of the images are similar to those in 8_1 containing an inhomogeneous halo. Several line-scratch-like defects are present, most likely “scratches” from sample preparation. This obviously complicates the local segmentation, as the scratch-like defects smear out the high-intensity lamellae along the defect’s direction.
- 24−C3: This batch contains brighter spots both within and outside of the lamellae regions; see Figure 4b.
- 24−C1: A combination of 30−1 with several branching lamellae. The images with typically high-intensity lamellae are plagued by the accompanying background and sometimes also branching.
- 22−B1: The batch data quality is pretty much similar to the 8_1.
- 30−2: Approximately 60% of the images have non-bright, somewhat grainy lamellae with a rather strong surrounding halo; see Figure 4a. The remaining 40% are somewhat grainy without the halo.
- 14_3_Novy: Approximately 50% of the images are rather grainy with lower intensities. Those with higher intensities exhibit branching/merging lamellae.

We note that the Otsu threshold provides the best result in this approach in the case of batches 8_1, 30−2, and 14_3_Novy. A decrease in absolute value of the relative difference $|\delta_{\bar{\sigma }:b}|$ is achieved within the approach (A2) by switching to the Yen threshold in the case of the other batches. The improvement ranges from fairly large 37% (in the case of the batch 30−1) through to small 14% (24−C1 and 22−B1) to none (8_1, 14_3_Novy and 30−2). This clearly shows that there is no “sweet” spot combination of settings that works for all the microstructure images very well.

The detailed analysis presented above implies that the higher deviation of the code-produced values with respect to the manually processed results clearly corresponds to images not containing bright regular lamellae well separated from the dark background not containing any halos.

It is possible that the performance on these data could be improved by altering the processing steps described in Section 2.4, namely, removing the global thresholding, step (S3), and adding local thresholding into the single measuring circle processing (currently step (S5)) as its first sub-step. However, suppression of the deviations due to this improvement might only be partial in the case of batches 8_1 and 24−C3. Both batches contain variations of (average local) intensity in the ROI; the former is characterized by an intensity gradient “across” lamellae, and the latter contains bright spots.

We have adapted the MATLAB DFT code from Ref. [4] for comparison with the CLM approach. In order to avoid coding in the flexibility in change of magnification (and pixel size), we have used it on 338 of the images with magnification 30,000× and a simple naming convention. This restriction minimizes the scope of necessary adjustments to the MATLAB code to a reasonable minimum, namely, cutting out the data bar and looping over different directories, each containing a single batch.

The DFT code provides apparent interlamellar distance by employing two processing methods. The best quantity to compare the two codes, Python CLM and MATLAB DFT, is the mean interlamellar distance $\bar{\sigma }$ with the mean across all measuring circles within a single image or batch average of these individual per-image values. We select the former approach.

Let us start the comparison by making some general comments. The DFT-based approach fails to a varying degree in the case of two images containing mostly a single, very regular pearlite colony. It fails completely, requiring more points to process in the Fourier-transformed domain in the case of an image with a corner-located irregularity (some 4% of the image) at the edge of the dominant pearlite colony. In the case of the other problematic image (from batch 1_2), the result of a pearlite colony with some lamellae having endpoints within the field of view, the first method yields a negative result. We believe that both these failures can be circumvented by lowering the magnification, thus increasing the number of pearlite colonies in the field of view. Apart from these two exceptions, the code performed without issues.

**Figure 8:**
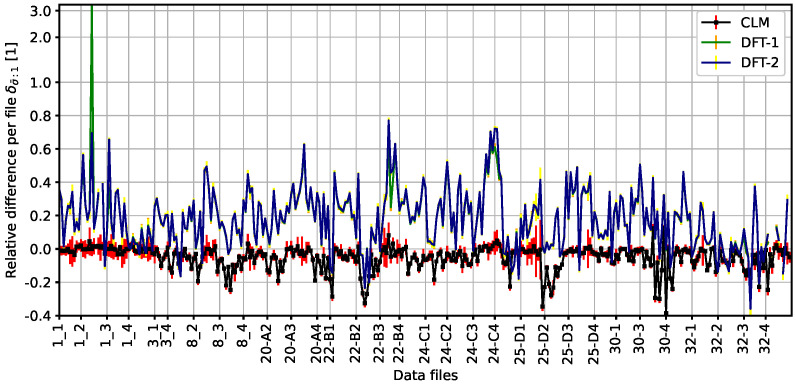
Comparing the relative difference $\delta_{\bar{\sigma }:1}$ of the two methods of the Matlab code, labeled by DFT−1 (color: green data with orange error bars) and DFT−2 (color: navy blue data with yellow error bars) with respect to the manual data across the 338 files considered. The corresponding result for the CLM code (color: black data with red error bars) presented here is added. Labeling all files would make the *x* tick labels incomprehensible; hence, the label of the *x* axis indicates only the start of a new batch. As a result, the width of the corresponding/following grid segment indicates the number of files. The scale of *y* axis is the so-called “symlog”, combined from a linear scale within the range [−1,1] and a logarithmic scale outside of the linear range, in order not to suppress the results within the linear range by outlier results.

In order to compare the MATLAB DFT-based code results with those here presented, the interlamellar distance is scaled from apparent to random using Equation (1). Then, we calculate the relative difference $\delta_{\bar{\sigma }}$ introduced in Equation (4) and plot the results in Figure 8. As the relative difference is per file, we use more precise notation of $\delta_{\bar{\sigma }:1}$ in accordance with Equation (3).

The error bars assigned to the two methods of the MATLAB code are derived solely from the standard deviation of the interlamellar distance from the manually processed dataset for all measuring circles within each given file. The MATLAB DFT-based code tends to underestimate the interlamellar distance when compared to the manually processed data; on average, the underestimation is by some 15%. The value is estimated from fitting a multiplicative scale and minimizing the deviation with respect to the manually processed data using the SciPy [12] non-standard Python library. The largest observed deviation occurs in the case of method 1 and batch 1_2; the corresponding file contains a single pearlite colony with fairly uniform distribution apart from several interrupted or merging lamellae. This is where the DFT-based code, by design, does not perform well, as its above-discussed failures indicate. The fact that the results from the Python code match the manual processing better than the MATLAB DFT-based code is not surprising. Indeed, the dataset was used for fine-tuning configurable parameters of our code and the MATLAB DFT-based code implements a different method.

Compare the different types of results corresponding to the Python CLM code. The data for deviation both per batch $\delta_{\bar{\sigma }:b}$ (see Figure 7b) and per file $\delta_{\bar{\sigma }:1}$ (displayed in Figure 8) reveal that the statistical processing per batch averages out some outliers in the deviation per file. Consider the sample no. 1; although the individual per-file deviations may be larger, $\left|\delta_{\bar{\sigma }:1}\right|$ is less than 4.56%, and the per-batch deviation satisfies the stricter limit $\left|\delta_{\bar{\sigma }:b}\right|$ not exceeding 1.2%. We expect that a similar effect would also apply to the DFT-based code, thus making the differences less pronounced in a not-displayed equivalent of Figure 7b.

## 4. Conclusions and Outlook

We have presented a tool that partially automates the CLM method by counting the intersections for known positions of the measuring circles. This is achieved using simple image processing methods. It turns out that, naturally, the performance in the intersection count domain depends on the thickness and regularity/linearity of the lamellae and their intensity separation from the background. Despite this dependence, a suitable choice of parameters can ensure a reasonable deviation for all the samples in the benchmarking dataset available to us, as demonstrated in Figure 7.

Analysis of image batches with relative deviation of the mean values, $\delta_{\bar{\sigma }:b}$, exceeding 10% showed that these batches contain images of “lesser” quality—grainy, lower intensity lamellae, branching, and presumably surface scratches (see left-hand side of Figure 4a for an example) from sample preparation, etc. We have proposed a method to improve performance on these batches by switching from global thresholding to local thresholding in the ROI defined by the square tightly enclosing the measuring circle. This could be explored in future work and versions of the application. One of the desirable extensions would be to be able to assess the “quality” of a region and decide whether placing a circle there was, or is, favorable or it is not; this could help to provide suggestions for circle positions to be (partially) accepted or rejected by the end user.

Despite the more feature-rich image data with irregularities leading to lower performance, the current code already exhibits excellent performance on images with highly regular, well-separated lamellae. They correspond to sample no. 1 (batches from 1_1 to 1_4), and the deviation $\delta_{\bar{\sigma }:b}$ does not increase above 1.2%.

As “There ain’t no such thing as a free lunch” (TANSTAAFL) applies here as well, reaching optimal performance may require more than a single run. The suggested method is that the end user visually inspects the images to be processed, they split them into several classes, and then they test the performance on a suitable representative of each class for different configuration parameters. Processing of each image class may benefit from using (slightly) different parameters.

The above-demonstrated good-to-excellent performance for interlamellar distance is proof that this tool can be used to automate the CLM data processing. As a result, it takes the burden of tedious manual counting of intersections for all the imaged microstructures from its operator, leaving only the selection of the positions of the measuring circles. Of course, there is a much smaller price to pay to achieve the best performance, namely, the testing of the configurable parameters on a small subset of images.

## Data Availability

A small sample subset of the image pairs, both with and without circles, with the automatically detected circle position and results from both Python and MATLAB codes, is available upon reasonable request. Because we are in process of commercialization of the application, neither its source code nor compiled binary form will be disclosed beyond the description of the algorithm presented in this paper.

## Abbreviations

    The following abbreviations are used in this manuscript:

AI	Artificial intelligence
CBS	Concentric back-scattered (detector)
CLM	Circular line method
DFT	Discrete Fourier transformation
ETD	Everhart-Thornley Detector
GIMP	GNU Image Manipulation Program
GUI	Graphical user interface
ML	Machine learning
PyPI	Python Package Index
ROI	Region of interest
SEM	Scanning electron microscope

## Appendix A. Non-Standard Python Libraries Utilised

The following non-standard Python 3.11 libraries have been utilized in (preparation of) the CLM Python code presented in this paper. The below is extracted from the requirement file used to easily recreate development and build virtual environment. The inline comments indicate role of the library in the code

configobj == 5.0.9 # Configuration handling (store, load, verify)
easyocr == 1.7.2 # Auxiliary automatic detection of the circle positions
matplotlib == 3.10.0 # Plotting
mypy == 1.18.2 # Code linter, no functional use
numpy == 2.2.2 # Array code
opencv-python == 4.11.0.86 # Image operations, incl. segmentation and overlays
pandas == 2.2.3 # Storing|Loading data in|from DataFrames (e.g., named columns)
pandas-stubs # Related to code linter
pyinstaller == 6.12.0 # Building resulting binary
pylint # Code linter, no functional use
pytesseract == 0.3.13 # OCR
scipy == 1.15.1 # Statistical processing
scipy-stubs # Related to code linter
scikit-image == 0.25.1 # Image thresholding

The libraries and their description can be found at Python Package Index [13] (PyPI) or at the individual project webpages linked at webpages of these individual packages at PyPI.

## Appendix B. Key Parameters of the CLM-Code Processing

The following relevant parameters have been used in order to acquire the CLM code results presented in Section 3.

[Configuration]
conffile = /tmp/pearlite_data.ini

[Images]
files = <path to file(s)>
device = Test # Select from a selection

[Databar]
location = bottom
height = -60

[Size pixel]
method = auto-OCR
value = 999.0 # ignored if method == auto-OCR|meta

[Magnification]
method = auto-OCR
value = 999.666 # ignored if method == auto-OCR|meta

[Lamellae]
intensitylow = False

[Processing]
debug = False
logfile = /tmp/pearlite_data.log
loglevel = debug

[Circles]
radius = 1.0 # real-space radius of the circle; unit as pixel size
method = manual # manual|auto; re-use existing files, if any
count = 0
overlap = 0.0
thickness = 1 # Thickness of the edge of the circle
padding = 5 # Additional padding [px] of a square enclosing the circle

[Parameters]
threshold = Otsu (SKImage)
blurring = 1 # OpenCV’s blurring parameter

[Output]
full = full.csv # Full output file
statistics = stat.csv # Statistics overview file
stat_all = True
extension_image = png # Extension of output image(s)

Some parameters have an accompanying inline commentary added. The value of parameter files has been replaced with a mere indication of its meaning. The tests were run in a Linux operational system containing the folder /tmp/. Different threshold methods, parameter threshold, implemented in SciKit-Image [11] (its module filters), have been tested in the image pre-processing before segmentation.
